# Cell wall fucosylation in Arabidopsis influences control of leaf water loss and alters stomatal development and mechanical properties

**DOI:** 10.1093/jxb/erad039

**Published:** 2023-01-30

**Authors:** Paige E Panter, Jacob Seifert, Maeve Dale, Ashley J Pridgeon, Rachel Hulme, Nathan Ramsay, Sonia Contera, Heather Knight

**Affiliations:** Department of Biosciences, Durham University, South Road, Durham, UK; Department of Physics, University of Oxford, Parks Road, Oxford, UK; Department of Biosciences, Durham University, South Road, Durham, UK; School of Biological Sciences, University of Bristol, Bristol, UK; School of Biological Sciences, University of Bristol, Bristol, UK; Department of Biosciences, Durham University, South Road, Durham, UK; Department of Biosciences, Durham University, South Road, Durham, UK; Department of Physics, University of Oxford, Parks Road, Oxford, UK; Department of Biosciences, Durham University, South Road, Durham, UK; University of Essex, UK

**Keywords:** AFM, *Arabidopsis thaliana*, cell wall, cuticular ledge, elastic modulus, fucose, guard cell, *MUR1*, RGII, stomata

## Abstract

The Arabidopsis *sensitive-to-freezing8* (*sfr8*) mutant exhibits reduced cell wall (CW) fucose levels and compromised freezing tolerance. To examine whether CW fucosylation also affects the response to desiccation, we tested the effect of leaf excision in *sfr8* and the allelic mutant *mur1-1*. Leaf water loss was strikingly higher than in the wild type in these, but not other, fucosylation mutants. We hypothesized that reduced fucosylation in guard cell (GC) walls might limit stomatal closure through altering mechanical properties. Multifrequency atomic force microscopy (AFM) measurements revealed a reduced elastic modulus (*E*ʹ), representing reduced stiffness, in *sfr8* GC walls. Interestingly, however, we discovered a compensatory mechanism whereby a concomitant reduction in the storage modulus (*E*ʹʹ) maintained a wild-type viscoelastic time response (tau) in *sfr8*. Stomata in intact leaf discs of *sfr8* responded normally to a closure stimulus, abscisic acid, suggesting that the time response may relate more to closure properties than stiffness does. *sfr8* stomatal pore complexes were larger than those of the wild type, and GCs lacked a fully developed cuticular ledge, both potential contributors to the greater leaf water loss in *sfr8*. We present data that indicate that fucosylation-dependent dimerization of the CW pectic domain rhamnogalacturonan-II may be essential for normal cuticular ledge development and leaf water retention.

## Teaser

We show that fucose-dependent pectin cross-linking is required for normal stomatal development and control of leaf water loss. Reduced guard cell wall stiffness in a fucosylation-defective mutant did not influence stomatal closure.

## Introduction

Plant cells are surrounded by a cell wall (CW) which provides rigidity whilst allowing growth, and affords protection from the external environment. CWs are typically comprised of cross-linked cellulose microfibrils embedded in a matrix of hemicelluloses such as xyloglucans (XGs), xylans, and mannans (Brett and Waldron, 1990), and pectin, including homogalacturonan (HG), rhamnogalacturonans-I and -II (RG-I and -II), arabinans, galactans, and arabinogalactans (Caffall and Mohnen, 2009) and embedded proteins (Albenne *et al.*, 2014). Pectins can be cross-linked via borate diester linkages between RG-II domains (Kobayashi *et al.*, 1996; O’Neill *et al.*, 1996), by arabinan chains within RG-I domains, or via Ca^2+^ ions between HG chains (Jarvis, 1984). Mutants in CW composition have provided useful tools to probe the function of the CW in a variety of processes. The *murus* (*mur*) mutants were identified in a screen for Arabidopsis mutants with altered CW polysaccharide composition (Reiter *et al.*, 1997). *mur1* mutants have very low levels of CW fucose, due to a mutation in the gene encoding the enzyme GDP-mannose 4,6-dehydratase, which catalyses the *de novo* production of cellular fucose (Bonin *et al.*, 1997). A lack of fucosylation on the side chain A of the pectic domain of RG-II results in reduced borate cross-linking of RG-II chains in the CWs of *mur1* mutants, leading to a number of effects including reduced CW tensile strength and changes to growth (O’Neill *et al.*, 2001; Ryden *et al.*, 2003). Recently, we cloned *SENSITIVE-TO-FREEZING-8*, originally identified in a forward genetic screen for mutants with reduced freezing tolerance (Warren *et al.*, 1996). We discovered that *sfr8* was an allelic mutant of *mur1-1* and *mur1-2*, implicating the CW, and pectin specifically, in the tolerance of adverse temperature stress (Panter *et al.*, 2019). This adds to a growing wealth of literature implicating the CW in the plant’s response to and defence against its environment (Houston *et al.*, 2016).

The composition of the CW has particular relevance to cells with specialized mechanical function, including stomatal guard cells (GCs). Stomata are small pores in the leaf epidermis that allow gas exchange for the metabolic processes of photosynthesis and respiration, as well as exchange of water vapour in the process of transpiration. A pair of GCs surround the stomatal pore, which is opened via the synthesis of osmolytes and influx of solutes into the GC, triggering uptake of water to cause an increase in turgor. GC movements are affected by potassium ion (K^+^) influx; artificially increasing this influx across the plasma membrane accelerates movements (Papanatsiou *et al.*, 2019). Conversely, the efflux of ions, and thus water, results in flaccid GCs and stomatal closure (Blatt, 2000). GCs respond to a variety of environmental parameters including light, water availability, temperature, CO_2_ concentration, humidity, vapour pressure deficit, and exposure to pathogens. These signals fine-tune stomatal movements to optimize the balance of CO_2_ uptake with water loss (Willmer and Fricker, 1996; Lawson and Matthews, 2020). Stomatal movements are associated with changes in GC morphology as a result of the interaction between turgor pressure and CW mechanical properties. Guard CWs (GCWs) have a specific polysaccharide composition to accommodate changes in turgor pressure, and altering this composition can affect stomatal function (Jones *et al.*, 2003; Liang *et al.*, 2010; Amsbury *et al.*, 2016; Rui and Anderson, 2016; Huang *et al.*, 2017; Rui *et al.*, 2017). GCWs are enriched with pectin, containing a high level of unesterified HG (Amsbury *et al.*, 2016). They are reinforced by radially aligned cellulose microfibrils that direct the increase in cell volume to a lengthening of the GC, resulting in an increase in pore area (Meckel *et al.*, 2007). In this study, we examine the effect of the *sfr8* mutation on leaf water retention and explore the effects that fucosylation-related changes to the GCW have on stomatal function and morphology.

## Materials and methods

### Plant material and growth conditions

This study used Col-0 wild-type Arabidopsis, *mur1-1* and *mur2* [N6243 and N8565 respectively; Nottingham Arabidopsis Stock Centre (NASC)], *sfr8* and *MUR1*-complemented *sfr8* (*sfr8-C*) plants (Panter *et al.*, 2019), the *bor1-3bor2-1* double mutant (Miwa *et al.*, 2013), *fut4* (Tryfona *et al.*, 2014), *cgl1-2* (Frank *et al.*, 2008), and *gpat4 gpat8* (Li *et al.*, 2007). Seeds were initially sown on 0.8% agar with half-strength Murashige and Skoog (MS) growth medium and grown at 20 °C. Except where stated otherwise, seedlings were transferred to jiffy plugs for further growth to the rosette stage, at 20 °C; 12 h light/12 h dark; 150–200 µmol m^–2^ s^–1^ light [phosynthetically active radiation (PAR) 400–700 nm], as previously described (Panter *et al.*, 2019). For boron supplementation, seeds were sown on 0.5× MS supplemented with 0.1 mM potassium tetraborate tetrahydrate or 0.1 mM potassium chloride (KCl, controls). pH was adjusted to 5.8 with 0.1 M KOH. After transfer to peat plugs, plants were grown as above but watered twice per week with dH_2_O containing 0.1 mM potassium tetraborate tetrahydrate or 0.1 mM potassium chloride.

For leaf disc stomatal measurements, plants were grown on soil as previously described (Pridgeon and Hetherington, 2021).

### Leaf water loss

Water loss from individual leaves excised from 5-week-old plants was assessed as we have done previously (Lee *et al.*, 2021). Briefly, individual fully expanded leaves were excised from plants that had been maintained at 100% humidity for 16 h prior to experimentation to increase stomatal opening. Leaves were weighed in individual weigh-boats immediately after excision using a Kern PFB precision balance 0.001 g capable of measuring to the nearest 1 mg. Weigh-boats with leaves with the abaxial side uppermost were maintained on the laboratory bench in between measurements, at ~21 °C and under humidity levels of between 45% and 55%. Each experiment was performed on three separate occasions, each using 6–7 leaves per genotype, each leaf from a separate plant.

### Toluidine blue staining

Cuticle permeability was assessed using the method developed by Tanaka *et al.* (2004), with some modifications. Briefly, true leaves were carefully removed from plants during the dark cycle (to reduce stomatal opening) and placed adaxial or abaxial side upwards on an MS agar Petri plate. A 2 µl droplet of filter-sterilized 0.025% toluidine blue O (TBO) was placed on each leaf surface and the Petri plate lid replaced for 20 min. After this, leaves were gently but thoroughly washed by swirling in 500 ml of deionized water. Leaves were placed on a new agar plate after washing and observed with the use of a Leica M80 stereo microscope and ×5 lens.

### Stomatal aperture and size measurements

For measurements of stomatal complexes and pore apertures, epidermal peels from the abaxial side of the leaf were obtained from 4-week-old rosette plants as previously described (Gonzalez-Guzman *et al.*, 2012). Briefly, epidermal peels were obtained ~1 h after dawn and incubated in 10 mM MES/KOH, 50 mM KCl, pH 6.2 at 20 °C, 150–200 µmol m^–2^ s^–1^ light for 2 h. Peels were transferred to a microscope slide and imaged using a Leica light microscope at ×20 magnification. The experiment was performed 2–3 times, each using three epidermal peels per genotype, with 15 stomata measured per peel. Images were analysed using ImageJ. Data were analysed by a one-way ANOVA followed by a post-hoc Tukey test. To measure dynamic changes in aperture in response to a closure stimulus, leaf discs (4 mm in diameter) were harvested from plants 2 h after dawn and incubated in buffer as above at 22 °C for 2 h in 120 µmol m^–2^ s^–1^ light. Stomata were imaged using a Leica DMI6000 B inverted microscope and apertures measured 0, 10, 30, and 60 min after transfer to buffer containing 10 µM abscisic acid (ABA). The experiment was repeated three times with a total of 30 stomata from three individual plants measured. Data were analysed using a two-way ANOVA. All images were analysed using ImageJ (FIJI).

### Atomic force microscopy (AFM)

All experiments were performed with the Cypher ES (Oxford Instruments Asylum Research, CA, USA). The microscope was operated in contact resonance with feedback on the deflection of the cantilever, as it is simultaneously oscillated at the first eigenmode using photothermal actuation as described previously (Seifert *et al.*, 2021). The cantilever (Olympus, OMCL-AC160TSA) had a nominal spring constant of *k* ~26 N m^−1^, a resonance frequency in MS medium of ~140 kHz, and a quality factor (Q) of ~9. The cantilever was calibrated using the method of Sader *et al.* (2016). In our previous study, we investigated in detail the validity of the frequency in the particular case of the plant CW (Seifert *et al.*, 2021). The scan rate was 2.44 lines s^–1^ with 255 pixels per line, corresponding to a pixel size of 78 nm. The free amplitude was set to *A*_1, far_ ~14 nm using a blue laser with a power of *P*_blue_=8 mW for photothermal activation. The amplitude in contact with the sample (*A*_1_) was A_1, near_ ~4 nm with a setpoint of the deflection of 0.3 V, corresponding to an indentation depth of ~300 nm. The exact drive frequency was re-tuned before each scan, and at the same time the phase far from the surface was set to ϕ_1, far_=90°. At the end of each scan, a quasi-static indentation curve was obtained in the centre of the image to produce a calibration curve for the amplitude and phase near the sample and the free amplitude, as required by the method of Seifert *et al.* (2021). A brief summary of this workflow is shown in Supplementary Fig. S1.

Cotyledons from 5-to 6-day-old seedlings were gently attached to the probe holder of the microscope (15 mm diameter metal plates) with Hollister 7730 medical adhesive spray, abaxial side facing up. Due to the unevenness of true leaf surfaces, it was necessary to use cotyledons for this assay. The cotyledon was placed inside a Petri dish with wet tissue to maintain hydration while the glue set. A drop of half-strength MS medium was placed on the sample for AFM imaging. AFM data were analysed with Python3.5 (https://www.python.org/) as described previously (Seifert *et al.*, 2021; the code is available at https://github.com/jcbs/ForceMetric). Data were analysed using a two-sample *t*-test.

### SEM imaging

Leaf sections (~2 × 6 mm) were fixed in 2% paraformaldehyde, 2.5% glutaraldehyde in 0.1 M sodium cacodylate buffer pH 7.4 for 1.5 h. After rinsing with 0.1 M sodium cacodylate buffer, fixation continued in 1% osmium tetroxide in 0.1 M sodium cacodylate buffer pH 7.4 for 2 h. The leaves were then dehydrated though an ascending series of alcohol. The leaves were critically point dried, attached to silicon chips, and coated with 5 nm of platinum before viewing with an S5200 FESEM at 10 kV (Hitachi, Japan). Approximately 40 images of individual stomata from four plants per genotype were taken.

## Results

### Leaf water loss is greater in the fucose-deficient mutant *sfr8* than in the wild type

**Fig. 1.**
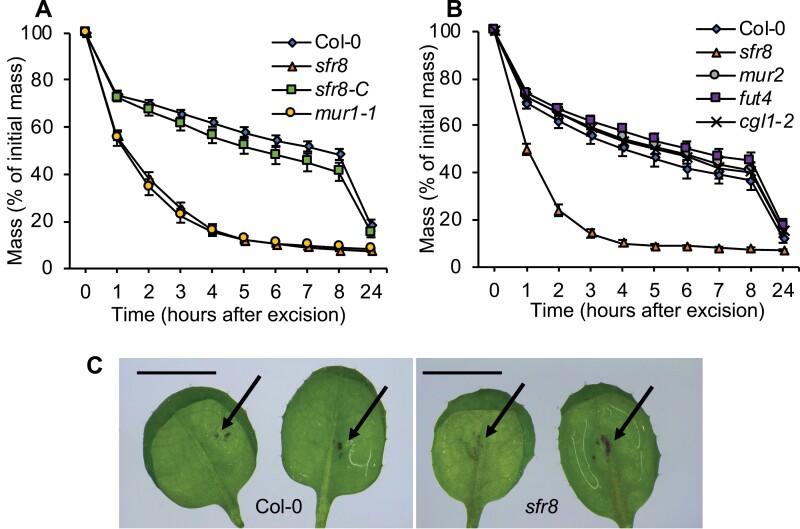
Fucose-deficient mutants lose water rapidly after leaf excision. (A, B) Leaf mass as a percentage of initial mass after leaf excision in the wild type (Col 0) and mutants with altered cell walls. Five-week-old plants of Col-0 and the mutants were maintained in a water-saturated environment for 16 h before experiments were conducted. Leaves were transferred to ~50% humidity and 21 °C, and weighed every hour for 8 h, then at 24 h. Error bars represent ±1 SE of (A) 21 or (B) 18 leaves from individual plants collected over the course of three separate experiments. (C) Cuticle permeability. A 2 µl droplet of 0.025% toluidine blue O was applied to the abaxial surface of leaves from 17-day-old plants. Images taken with a Leica IC90 E camera fitted to a Leica Stereo microscope using ×5 magnification show the typical range of staining patterns seen in either genotype after thorough washing in distilled water, and arrows mark the position of staining observed. These patterns are representative of those seen in four independent tests. Scale bar represents 5 mm.

To test whether *SFR8*/*MUR1* was required for the control of leaf water loss, mature rosette leaves were excised from plants and their water loss recorded on an hourly basis. Excised mature rosette leaves from *sfr8* and the allelic mutant *mur1-1* lost water more quickly than the wild type (Col-0) or an *sfr8 MUR1-*complemented line (*sfr8-C*) (Fig. 1A). Enhanced water loss was particularly evident in the first few hours after excision. These data indicated that the more rapid water loss in the two allelic mutants was genetically linked to *MUR1* and associated with reduced CW fucose levels. To gain insight into whether the reduction in a particular fucosylation event might be responsible for the phenotype, we tested leaf water loss in a set of fucosylation-related mutants. *mur2*, *fut4*, and *cgl1-2* all responded similarly to the wild type in the assay (Fig. 1B). *mur2* lacks XG fucosylation (Vanzin *et al.*, 2002) due to a mutation in the gene encoding fucosyl transferase 1 (*FUT1*). The *fut4* mutant lacks a fucosyltransferase specific to CW arabinogalactan proteins and active in leaves (Liang *et al.*, 2013), and *cgl1* mutants lack the ability to process N-linked glycan, a cell wall component that is fucosylated; therefore, *cgl1-2* lacks fucosylated glycoproteins (Frank *et al.*, 2008). Together these data eliminated some possible consequences of reduced fucose levels as the cause of the water loss phenotype we observed and suggested that impairment of a specific fucosylation even might be the reason for our observations.

Most water is lost from leaves either via stomata or by evaporation through the cuticle. We compared the cuticle permeability of *sfr8* with that of the wild type and a known cuticle-permeable mutant, *gpat4 gpat8*, which shows reduced cutin deposition due to lack of two glycerol-3-phosphate acyltransferase (GPAT) enzymes and, as a result, exhibits large increases in leaf water loss (Li *et al.*, 2007). Using a modification of an established method that monitors the penetration of TBO dye into internal tissues, we observed much greater penetration of a droplet of TBO applied to the adaxial surface into leaves of *gpat4 gpat8* after 20 min compared with the wild type or *sfr8* (Supplementary Fig. S2), with dark blue staining clearly visible in *gpat4 gpat8* as previously reported (Li *et al.*, 2007). Having established that our methodology was suitable to identify differences in cuticle permeability, we applied droplets of TBO to the abaxial side of wild-type and *sfr8* leaves. Examination under the microscope revealed a small amount of staining in both wild-type and *sfr8* leaves. *sfr8* leaves on average showed slightly more staining than those of the wild type, but no major differences were evident (Fig. 1C). We concluded that whilst *sfr8* leaves may have slightly more permeable cuticles than the wild type, any differences are relatively small and unlikely to account entirely for the large differences in leaf water loss.

Pectins act as adhesion molecules (Lord and Mollet, 2002), and cell adhesion mutants show rapid leaf water loss after excision (Bouton *et al.*, 2002). However, *sfr8* did not exhibit the attributes of an adhesion mutant; cotyledons appeared normal and not fused to each other or to hypocotyls (Supplementary Fig. S3A, B), and closer examination of dark-grown hypocotyls stained with propidium iodide revealed no abnormal cell arrangements or protuberances indicative of cell dissociation or sloughing off (Supplementary Fig. S3C, D), as observed in other cell adhesion mutants (Neumetzler *et al.*, 2012; Verger *et al.*, 2016). Therefore, we considered that the greater water loss associated with the leaves of plants with mutations in the *MUR1* gene could potentially be explained by altered stomatal distribution, size, or function.

### *sfr8* shows differences in stomatal complex size and aperture compared with the wild type

**Fig. 2.**
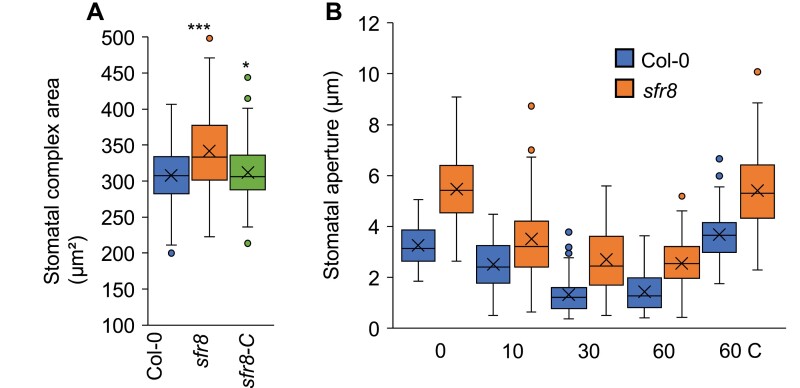
*sfr8* stomatal complex areas are greater than in the wild type but closure in response to ABA follows a similar pattern. The coloured boxes represent the interquartile range, the centre line is the median, x is the mean, and bars are the maximum and minimum values. (A) Stomatal complex area. Results are averages from two separate experiments each measuring three peels from separate plants with 10–15 stomata per peel. Data were analysed by two-way ANOVA followed by post-hoc Tukey test. Results shown represent significant difference from Col-0. *P<0.05, ***P<0.001. (B) Stomatal aperture measured from leaf discs of the wild type (Col-0) and *sfr8* after 0, 10, 30, and 60 min incubation in 10 µM ABA, and after 60 min without ABA treatment (60 C). Results are averages from three separate experiments each with *n*=30. Data were analysed by two-way ANOVA.

Neither stomatal density (Supplementary Fig. S4A) nor stomatal index (Supplementary Fig. S4B) differed significantly between Col-0 and *sfr8* plants, consistent with previous reports on a *MUR1* mutant, *scord6*, in which stomatal density was similar to that of wild-type plants (Zeng *et al.*, 2011). These data suggested that the increased water loss was not due to an increase in the number of stomata but they did not eliminate the possibility that the size of stomata might differ. We used epidermal leaf peels incubated in an opening buffer to assess stomatal complex size in the different genotypes. *sfr8* exhibited a significantly larger average stomatal complex area than Col-0 or *sfr8-C* stomata (Fig. 2A; *P*<0.001). Similarly, *mur1-1* stomata showed a significantly larger complex area than Col-0 (Supplementary Fig. S5; *P*<0.001).

### Dynamic stomatal behaviour in response to ABA is unaltered in *sfr8*

Under low humidity, wild-type stomata close in response to ABA to reduce water loss (Bauer *et al.*, 2013). Using intact leaf discs to view dynamic stomatal behaviour, we observed that stomatal aperture decreased in both Col-0 and *sfr8* in response to ABA within 10 min, reaching its lowest level within 30 min (Fig. 2B). However, the aperture was significantly greater for *sfr8* than for Col-0 (*P*>0.001), consistent with the larger stomatal complex size we had observed in *sfr8*. These data indicate that greater stomatal pore size may contribute to the greater water loss we observed, but showed that stomata of *sfr8* were able to respond to the drought hormone ABA to reduce pore aperture.

### Mechanical properties of the guard cell wall are altered by reduced cell wall fucose

Although our experiments showed that *sfr8* stomata could respond to the phytohormone ABA applied to whole-tissue leaf discs, they did not rule out the possibility that *sfr8* GCWs might have altered mechanical properties that could influence how they respond to natural desiccation signals. Using AFM, we assessed time-dependent viscoelastic mechanical properties (i.e. complex modulus) of *sfr8* and Col-0 GCWs. We used a multifrequency AFM technique that has been extended recently to correctly and quantitatively map two mechanical parameters in the CWs of living plants; the elastic modulus (*E*ʹ) and loss modulus (*E*ʹʹ) (Raman *et al.*, 2011; Cartagena-Rivera *et al.*, 2015). *E*ʹ quantifies the elastic mechanical energy stored in the sample (i.e. the elasticity) and is related to the displacement of the material, while *E*ʹʹ measures the density of the energy dissipated during deformation (i.e. the viscosity). This new approach is a contact resonance imaging method whereby the cantilever is permanently in contact with the sample and makes it possible to obtain measurements for every pixel on the image, allowing simultaneous structural observations as well as across-cell quantifications to be made (Seifert *et al.*, 2021). Unlike previous mechanical measurements made with more commonly used quasi-static indentation AFM methods, these measurements quantitatively reproduce the expected values of the CW. The discrepancy of previous AFM results from the expected values can be explained by the fact that in a quasi-static indentation AFM experiment, the bending modulus of the wall has a major contribution to the result, rather than the mechanical properties of the CW material itself. Our technique avoids this problem by imposing a 300 nm indentation to ensure a significant deformation of the CW and a contact radius of the pixel size (~80 nm), while the ~5 nm oscillations measure the actual material properties (discussed in detail in Seifert *et al.*, 2021). The cantilever observables (Supplementary Fig. S6; example measurements are shown in Supplementary Fig. S7) allow the derivation of *E*ʹ and *E*ʹʹ using the generalized Maxwell model, which is applicable for any linear viscoelastic material (Seifert *et al.*, 2021). The data processing workflow is illustrated in Supplementary Fig. S1.

**Fig. 3.**
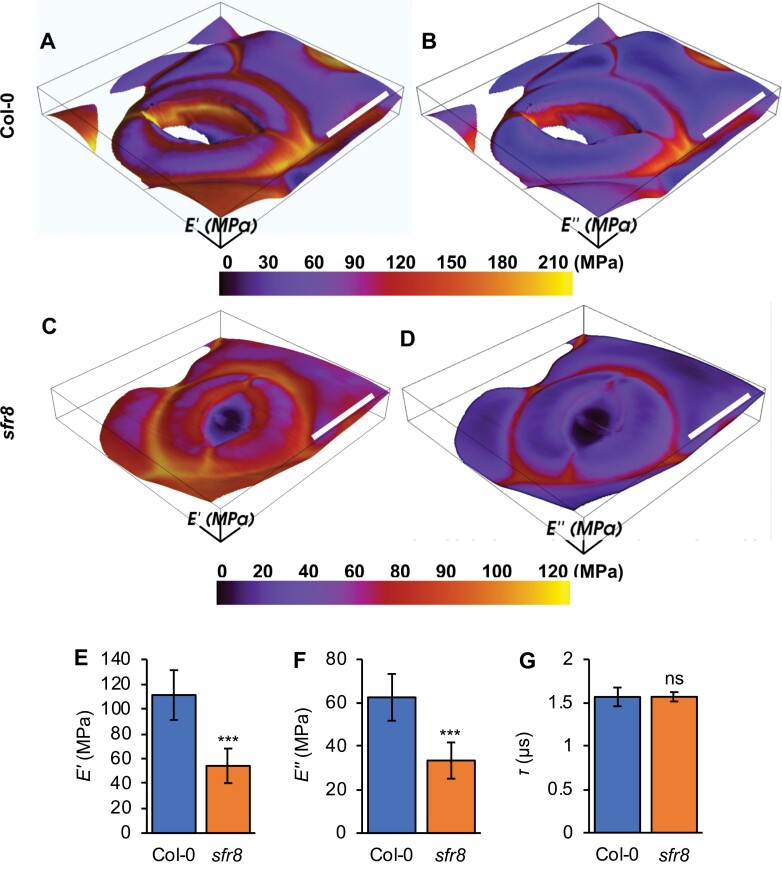
Multifrequency atomic force microscopy measurements show that viscoelastic properties differ in *sfr8* GC walls, but the time response remains the same. (A–D) Images of *Eʹ* and *E*ʹʹ obtained from multifrequency AFM using theory described in the text. The scan size is 30 × 30 µm. Scale bars=10 µm. (A, B) *E*ʹ and *E*ʹʹ values overlaid on the height image of the Col-0 stoma. (C, D) *E*ʹ and *E*ʹʹ values overlaid on the height image of the *sfr8* mutant stoma. (E) *E*ʹ and (F) *E*ʹʹ measured from stomata from Col-0 and *sfr8* cotyledons. (G) Time response, tau (τ), calculated as described in the Materials and methods. Data represent averages from six cotyledons each with 3–5 stomata measured. Error bars represent ±1 SE. Data were analysed by two-sample *t*-test. The results shown represent a significant difference from Col-0. ns, not significant, ***P<0.001.

We collected data from GCWs of open stomata of Col-0 and *sfr8* (Supplementary Fig. S7), from which we calculated *E*ʹ and *E*ʹʹ. Seedlings were used rather than mature leaves as the *z*-axis range in older leaves makes the measurements very challenging. Maps of *E*ʹ and *E*ʹʹ for two representative stomata showed quantitative differences between Col-0 (Fig. 3A, B) and *sfr8* (Fig. 3C, D). These findings indicate that *E*ʹ and *E*ʹʹ were both significantly lower in *sfr8* than in Col-0 GCWs (Fig. 3E, F; *P*<0.001), particularly at the polar regions, where stiffening is known to influence stomatal opening (Carter *et al.*, 2017). We note that our *E*ʹ maps qualitatively agree with Carter *et al.* (2017), including the stiffening at polar regions. We calculated the viscoelastic relaxation time response, tau (τ), of the GCW, and found that there was no significant difference between Col-0 and *sfr8* (Fig. 3G). This indicates that the time response of the GCW was unchanged in the mutant, appearing to concur with our observations made in leaf discs that *sfr8* and Col-0 stomata are equally capable of closure in response to the ABA treatments we performed. However, we cannot eliminate the possibility that the strikingly lower *E*ʹ and *E*ʹʹ might affect stomatal responses under some natural conditions that differ from our artificial ABA treatments.

**Fig. 4.**
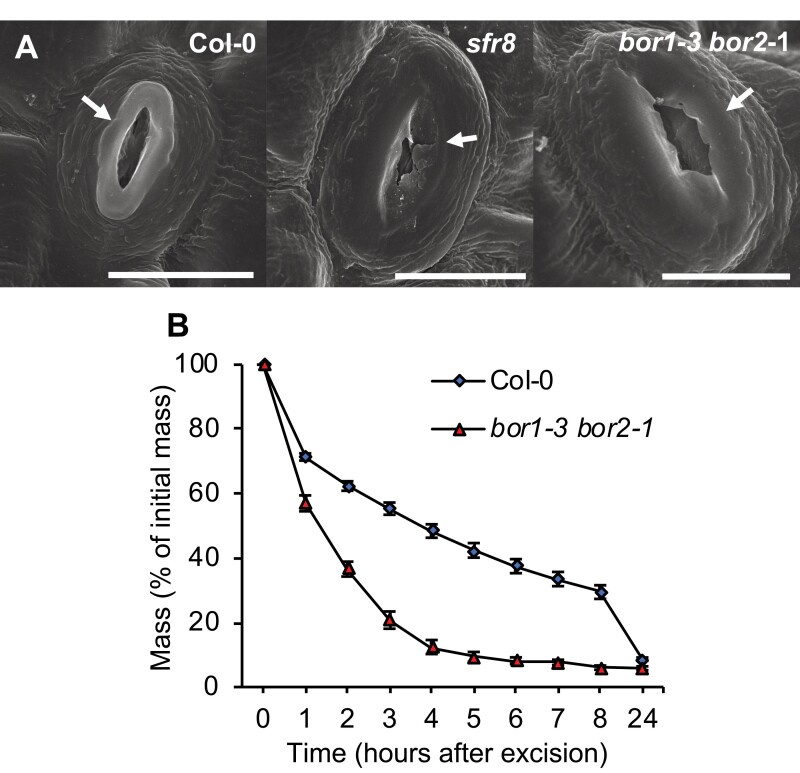
Cuticular ledge (CL) morphology and leaf water loss are affected in the *bor1-3 bor2-1* mutant. (A) SEM of wild-type (Col-0), *sfr8* and *bor1-3 bor2-1* mutant stomata. Scale bar=10 µm. The CL is indicated by a white arrow. Images are representative of samples from four separate experiments each with *n*=10. (B) Leaf mass as a percentage of initial mass after leaf excision in the wild type (Col-0) and a *bor1-3 bor2-1* mutant. Five-week-old plants of Col-0 and the *bor1-3 bor2-1* mutant were maintained in a water-saturated environment for 16 h before experiments were conducted. Leaves were transferred to ~50% humidity and 21 °C, and weighed every hour for 8 h, then at 24 h. Error bars represent ±1 SE of 21 leaves from individual plants collected over the course of three separate experiments.

**Fig. 5.**
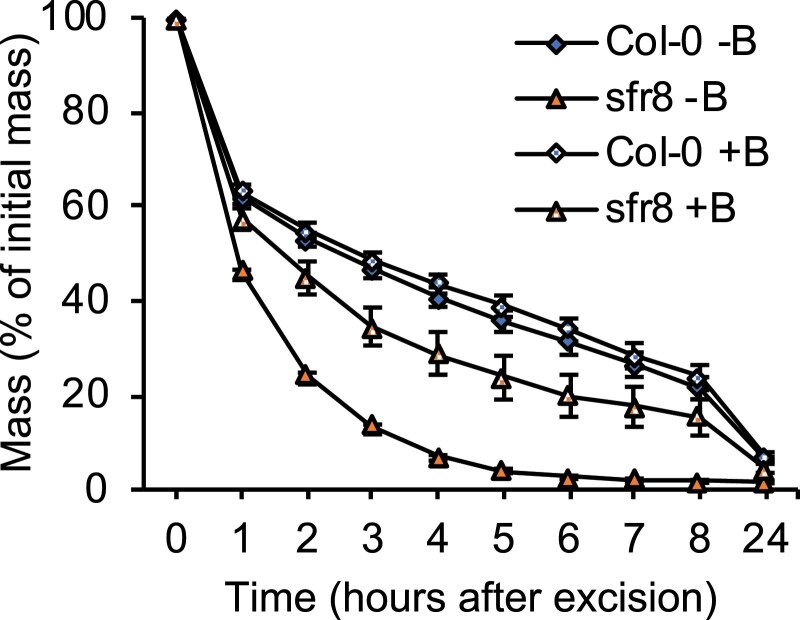
Boron supplementation throughout growth partially restores the water loss phenotype of *sfr8*. Leaf mass as a percentage of initial mass after leaf excision in the wild type (Col-0) and *sfr8* grown with (+B) or without (–B) boron supplementation. Five-week-old plants of Col-0 and *sfr8* grown under these conditions were maintained in a water-saturated environment for 16 h before experiments were conducted. Leaves were transferred to ~50% humidity and 21 °C, and weighed every hour for 8 h, then at 24 h. Error bars represent ±1 SE of 18 individual leaves from individual plants collected over the course of three separate experiments.

Previous studies have shown that in *mur1* mutants there is a reduction in the outer cuticular ledge (CL) surrounding the stomatal pore (Zeng *et al.*, 2011; Zhang *et al.*, 2019). SEM imaging confirmed that *sfr8* stomata also showed this altered morphology, exhibiting a reduced central ridge (Fig. 4A) causing the CL to lie flatter against the GCs. The CL is an extension of the GCW, and its structure has been linked with the prevention of water loss (Hunt *et al.*, 2017); other mutants in which stomatal CLs are lacking show increased transpiration rates (Li *et al.*, 2007; Macgregor *et al.*, 2008). This altered morphology of the stomatal pore might explain why leaves of *mur1-1* and *sfr8* lose water more rapidly than those of the wild type, whilst apparently retaining the ability to close normally. *sfr8* and *mur1-1* show reduced RG-II pectin dimerization due to their lack of fucose, and we speculated that this might be associated with the CL defect. Therefore, we examined stomata of the *bor1-3bor2-1* double mutant, which shows a reduction in the boron-mediated dimerization of RG-II pectin (Panter *et al.*, 2019), due to deficiencies in boron transport rather than any effect on fucose levels (Miwa *et al.*, 2013). *bor1-3bor2-1* also exhibited the same abnormal CL and central ridge morphology as *sfr8* (Fig. 4A), implicating RG-II cross-linking in normal CL development. We assessed leaf water loss in *bor1-3bor2-1* and found that like *sfr8* and *mur1-1*, *bor1-3bor2-1* mutants showed more rapid leaf water loss after leaf excision than Col-0 (Fig. 4B). To test further the possibility that RG-II boron cross-linking was responsible for the observed differences, we tested the effect of boron supplementation on the ability of *sfr8* to limit water loss. *sfr8* plants watered with potassium borate throughout growth showed a partial restoration of leaf water retention, while control plants watered with potassium chloride showed the response typical of the mutant (Fig. 5). Wild-type plants were not affected by the watering regime. Together, our results indicate that reduced CW fucose may affect leaf water loss through more than one mechanism including increases in GC size, possible mechanical effects, and via alterations in RG-II pectin cross-linking that bring about morphological changes to stomatal structure.

## Discussion

### A lack of CW fucosylation in *sfr8* results in a severe water loss phenotype

Previously we studied the response of a CW mutant, *sfr8*, to low temperature stress, and showed that it was sensitive to damage at freezing temperatures. An increasing number of reports reveal that the CW plays a role in defence against a variety of external stresses (Le Gall *et al.*, 2015; Houston *et al.*, 2016; Panter *et al.*, 2020). In this study, we examined the response of *sfr8* to another agronomically important abiotic stress condition, reduced water availability, and we found that leaves were very susceptible to water loss after excision, which could not be explained by any major changes in leaf cuticle permeability (Fig. 1). *sfr8* harbours a mutation in the *MUR1* gene, and the mutant shows severely reduced levels of CW fucose (Panter *et al.*, 2019), implicating CW fucosylation in the effect we observed. However, measurements on other fucosylation-related mutants indicated that it was unlikely that fucosylation of XG, N-linked glycan, or certain arabinogalactan proteins was required for water retention (Fig. 1B). Other substrates for fucosylation in the CW include RG-II pectin. Here, fucosylation of the pectic domain’s side chain A is a prerequisite for normal boron-mediated cross-linking between pectin monomers (Kobayashi *et al.*, 1996; O’Neill *et al.*, 2004). In contrast to the results observed with other fucosylation mutants (Fig. 1), the *bor1-3 bor2-1* mutant, which, like *sfr8*, is defective in boron cross-linking, did show abnormally high leaf water loss similar to *sfr8* (Fig. 4). Boron supplementation throughout growth is known to partially restore borate cross-linking of RG-II (O’Neill *et al.*, 2001), so we tested whether this would restore leaf water retention in *sfr8*. Our results showed that water loss from excised leaves of plants supplemented with boron was slower and less dramatic than seen in unsupplemented *sfr8* plants though still greater than in the wild type (Fig. 5). These two pieces of data provide good evidence that RG-II pectin cross-linking is likely to be important for leaf water retention. Pectins can act as cell adhesion molecules (Lord and Mollet, 2002), and we considered the possibility that lack of fucosylation on RG-II pectin rendered *sfr8* more prone to losing leaf water due to poor adhesion between cells, as is seen in the Quasimodo mutants (Bouton *et al.*, 2002). However, none of the usual morphological aberrations associated with defective cell adhesion (Bouton *et al.*, 2002; Verger *et al.*, 2016) was observed (Supplementary Fig. S3). Recent evidence suggests that boron-cross-linked pectin has better water-holding properties than non-cross-linked pectin, and this might have contributed to what we observed (Forand *et al.*, 2022). However, we predicted that the most likely primary reason for the effects we observed was an alteration in stomatal form or function.

### *sfr8* guard cell walls show reduced stiffness but reduced viscosity compensates for this

It is well known that the nature of the CW influences its mechanical properties (Peaucelle *et al.*, 2012), and various CW compositional changes have been shown to alter the mechanical function of guard cells in response to opening and closure signals (Jones *et al.*, 2003; Amsbury *et al.*, 2016; Rui and Anderson, 2016; Carroll *et al.*, 2022). Pectins play an important role in regulating CW stiffness, and recent studies have highlighted the influence of changes to pectin content on GC function, through modifying CW stiffness (Chen *et al.*, 2021; Carroll *et al.*, 2022). In one of these studies, a combination of computational modelling and empirical genetic manipulation showed that modifying the arabinan chain content of pectins reduced GCW stiffness, allowing the attainment of greater final stomatal pore apertures (Carroll *et al.*, 2022). Previously, mutants lacking pectin methylesterases (PMEs), which control the degree of methylesterification and subsequent cross-linking of HG chains, showed altered GC dynamics (Amsbury *et al.*, 2016; Huang *et al.*, 2017). Considering this growing body of evidence, we speculated that altering RG-II pectin cross-linking might also change GCW mechanical properties and impact upon stomatal opening and closure. Reduced stiffness in CWs of *mur1* hypocotyls has previously been described (Ryden *et al.*, 2003; Abasolo *et al.*, 2009), but measurements have not been made on GCWs. Therefore, we investigated the viscoelastic properties of CWs in *sfr8* GCs, using AFM. Because the presence of the cuticle makes AFM challenging on mature leaves (Rui *et al.*, 2018) and there are issues working with the extended *z*-axis scale in uneven leaf surfaces, seedling cotyledons make a more amendable system for plant CW AFM measurements (Bringmann and Bergmann, 2017; Robinson *et al.*, 2017).

Plant CWs are viscoelastic standard linear solids. Viscoelasticity is a combination of recoverable elastic deformation and time-dependent dissipative viscous deformation. CWs do not respond instantaneously to deformation, instead responding with a time delay that is related to the way in which the material dissipates energy as it is deformed at a given speed. The characteristic time delay of a viscoelastic material is related to the capacity to dissipate energy which can be quantified with the loss modulus (*Eʹʹ*, a measure of viscosity), whilst the elastic properties of the material can be measured with the elastic modulus (*Eʹ*, a measure of elasticity or stiffness). It was demonstrated recently that both *Eʹ* and *Eʹʹ* can be measured accurately at the nanoscale with multifrequency AFM (Seifert *et al.*, 2021). Previous work has correlated time responses used with this AFM technique with mechanical responses in the whole organ/plant on the second/minute time scales, discussed in detail in Seifert *et al.* (2021). Using this technique, in the present study we found that not only is *Eʹ* lower in *sfr8* GCWs than in those of Col-0, but *Eʹʹ* is also reduced (Fig. 3). Therefore, although lack of fucosylation leads to reduced stiffness of the *sfr8* GCW, the CW can compensate for this with a reduced capacity to dissipate energy as it is deformed. This ­compensatory mechanism means that the time response (tau, τ) of the CW does not differ significantly in the mutant at the second and minute time scales probed in our experiments, meaning that the CW material responds to deformation with the same time delay. At first sight, it seems counter-intuitive that an oscillation frequency over 100 kHz would probe mechanical properties that are relevant to the time scales of the opening and closing of the stomata. However, the hierarchical nature of the structure of the CW (which is a confined polymer nanocomposite network) can be invoked to understand this correlation. It has been shown that long-term relaxation times of larger scales depend on short-term relaxations at smaller scales; in the case of polymeric materials, this is related to the radius of gyration of the polymer (R_g_). Our AFM images of *Eʹ* and *Eʹʹ* reveal the contribution of polymer physics effects to the opening and closing of the stomata, but do not contain information on slower, biochemical processes that may happen at slower time scales. Our results qualitatively agree with previous published reports (Carter *et al.*, 2017) of elastic modulus *Eʹ* using static nanoindentation techniques in stomata. Whilst it cannot be assumed that identical results would be obtained had it been possible to do these experiments on stomata from more fully developed leaves rather than cotyledons, our measurements represent interesting observations about the differences in GCWs of the wild type and *sfr8*. Further work would be required in order to distinguish whether reduced fucosylation affects GCW mechanical properties via changes in RG-II cross-linking or by some other mechanism.

### Altered viscoelastic properties in *sfr8* guard cell walls do not influence ABA-induced closure in intact leaf tissues

When we tested the response to ABA in intact leaf discs, we observed no failure of *sfr8* stomata to respond to and close after ABA treatment (Fig. 2B), suggesting that under our experimental conditions, RG-II cross-linking might not play a role in stomatal closure dynamics. However, *sfr8* apertures were consistently greater than in the wild type, in line with the larger size of *sfr8* stomatal complexes (Fig. 2A). The compensatory mechanism described above that maintains the wild-type time response (tau) in *sfr8* GCWs might explain why, contrary to what we expected, the loss of stiffness of *sfr8* GCWs did not appear to affect the dynamic response of stomata to ABA. Other mutations that alter GCW pectin and its potential to cross-link and stiffen the wall do modify the GC response to ABA (Wu *et al.*, 2022). However, it should be borne in mind that our mechanical measurements are a snapshot of open stomata, and further analysis of time-dependent CW mechanics during opening and closure may well reveal an impact under particular conditions under which the CW is not able to compensate for reduced stiffness with reduced viscosity. Therefore, whilst we saw no difference in the response to ABA in intact leaf discs of *sfr8*, it is possible that under natural desiccation conditions the altered viscoelastic properties *sfr8* GCWs might affect their closure properties in a way that could explain the leaf water loss data we recorded.

### Morphological effects on stomata

The reduced CL we observed in *sfr8* plants, and which has been described previously in the *MUR1* mutant *scord6* (Zhang *et al.*, 2019), may contribute to increased water loss via the stomata. The CL is thought to contribute to the prevention of water loss from stomata as well as tilting the GCs, assisting with opening and closure of the pore (Willmer and Fricker, 1996; Kozma and Jenks, 2007). We discovered that the *bor1-3bor2-1* double mutant, which, like *sfr8*, has reduced RG-II cross-linking within the CW (Miwa *et al.*, 2013; Panter *et al.*, 2019), displayed similar CL morphology and leaf water loss to *sfr8*. This suggests that the structure of the CL may be reliant on fucosylation-dependent RG-II cross-linking specifically. The greater leaf water loss we observed in *sfr8* may be a consequence of larger pore size and reduced development of the CL. We saw no evidence that greater water loss was due to any differences in stomatal frequency, although our measurements were made on the abaxial surface of leaves and it should be noted that frequencies of stomata have been seen to be much lower on the adaxial side due to differences in the timing of stomatal precursor cell formation (Geisler and Sack, 2002). However, there is no reason to suppose that a lack of fucosylation would affect abaxial and adaxial stomatal development differently.

### Conclusion

To summarize, in this study we have shown that reduced CW fucosylation, potentially via its impact on RG-II cross-linking, resulted in larger GCs with altered CL morphology, both or either of which might account for increased leaf water loss from stomata. Despite the reduced *Eʹ* (which is expected for materials with reduced cross-linking) observed in *sfr8* GCWs, we saw no evidence of a mechanical failure of *sfr8* GCs to close in response to ABA treatment of leaf discs. Our direct observation of decreased GCW stiffness in *sfr8* was accompanied by a reduction in viscosity, suggesting the GCs can compensate for altered CW structure and mechanical properties to maintain cell shape and time response of the CW material. Further investigation would be necessary to ascertain if the changes to the time-dependent mechanical properties we observed in *sfr8* might impact upon GC dynamics under other dynamically changing environmental conditions.

## Supplementary data

The following supplementary data are available at *JXB* online.

Fig. S1. Processing flowchart including the experimental setup and calibration, and summary of assumptions necessary for calculating *Eʹ* and *Eʹʹ.*

Fig. S2. Penetration of toluidine blue stain is very much lower in leaves of both the wild type and *sfr8* than in *gpat4gpat6*.

Fig. S3. *sfr8* seedlings do not show obvious signs of cell adhesion defect.

Fig. S4. Stomatal distribution is unaltered in *sfr8*.

Fig. S5. Stomatal complex size in *mur1-1*.

Fig. S6. Schematic representation of the multifrequency atomic force microscopy (AFM) technique used in this study.

Fig. S7. Multifrequency AFM (with feedback on deflection and first harmonic actuated) images.

erad039_suppl_Supplementary_Figures_S1-S7

## Data Availability

All data supporting the findings of this study are available within the paper and within its supplementary data published online. Novel plant material (*sfr8*-complemented line) will be distributed upon request by HK.

## Abbreviations

AFM: atomic force microscopy
CL: cuticular ledge
CW: cell wall
GC: guard cell
GCW: guard cell wall
HG: homogalacturonan
*mur1*: *murus1*
RG-II: rhamnogalacturonan-II
*sfr8*: sensitive to freezing-8
XG: xyloglucan
