# Supplementary material for: Cell wall fucosylation in Arabidopsis influences control of leaf water loss and alters stomatal development and mechanical properties
Source: J Exp Bot. 2023 Jan 30;74(8):2680–91. doi: 10.1093/jxb/erad039 (PMC10112686; doi:10.1093/jxb/erad039)
Supplement: erad039_suppl_Supplementary_Figures_S1-S7 [file erad039_suppl_supplementary_figures_s1-s7.pdf]

Supplementary Materials. Panter *et. al.*

Fig.S1: Processing flowchart including the experimental setup and calibration, and summary of assumptions necessary for calculating E' and E''
Fig.S2: Penetration of toluidine blue stain is very much lower in leaves of both WT and *sfr8* than in *gpat4gpat6*.
Fig.S3: *sfr8* seedlings do not show obvious signs of cell adhesion defect.
Fig.S4: Stomatal distribution is unaltered in *sfr8*.
Fig.S5: Stomatal complex size in *mur1-1*.
Fig.S6: Schematic representation of the multifrequency atomic force microscopy (AFM) technique used in this study, based on (Seifert *et. al*., 2021).
Fig.S7: Multifrequency AFM (with feedback on deflection and 1st harmonic actuated) images.

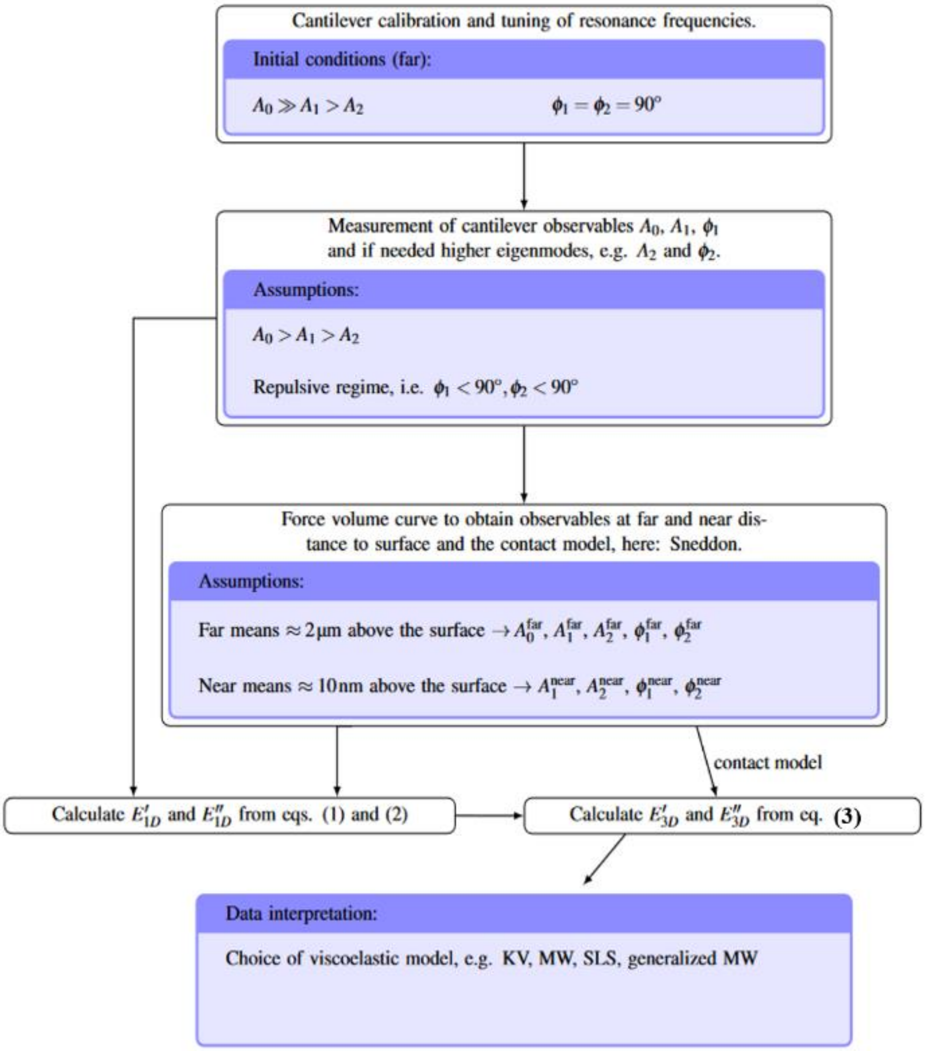

Fig.S1: Processing flowchart including the experimental setup and calibration, and summary of assumptions necessary for calculating E' and E'', using the procedure explained in detail in Seifert et al. 2021. (https://doi.org/10.1016/j.actbio.2020.12.010). In the figure KV, stands for Kelvin-Voigt model, MW is the Maxwell model, SLS is the standard linear solid. Reprinted from Acta Biomaterialia volume 121, Seifert J, Kirchhelle C, Moore I, Contera S. Mapping cellular nanoscale viscoelasticity and relaxation times relevant to growth of living Arabidopsis thaliana plants using multifrequency AFM. pp 371–382, Copyright (2021), with permission from Elsevier.

After calibration of the cantilever, the cantilever observables are measured. Then to be able to use the theory described in (Seifert 2021) we obtain the cantilever observables far and near the sample as outlined in the flowchart. Then we are able to calculate the 1-dimensional (1D) E' and E'' using the equations below:

$$F_0 = k_c A_0 = E_0(t)\delta_0$$

$$E' = F_{\text{osc}}\left(\frac{\cos(\phi_1)}{A_1} - \frac{\cos(\phi_{1,\text{near}})}{A_{1,\text{near}}}\right) \quad (1)$$

$$E'' = F_{\text{osc}}\left(\frac{\sin(\phi_1)}{A_1} - \frac{\sin(\phi_{1,\text{near}})}{A_{1,\text{near}}}\right). \quad (2)$$

Then using the contact model (in this case Sneddon, see again Seifert, 2021) we obtain E_0 and then we can calculate the 3-dimensional E' and E'' (3D) using the following equations:

$$E'_{3D} = \frac{E'_{1D}}{2}\sqrt{\frac{1-\nu^2}{\tan(\alpha)}\frac{\pi}{2}\frac{E_0}{F_0}} \quad (3)$$

$$E''_{3D} = \frac{E''_{1D}}{2}\sqrt{\frac{1-\nu^2}{\tan(\alpha)}\frac{\pi}{2}\frac{E_0}{F_0}}.$$

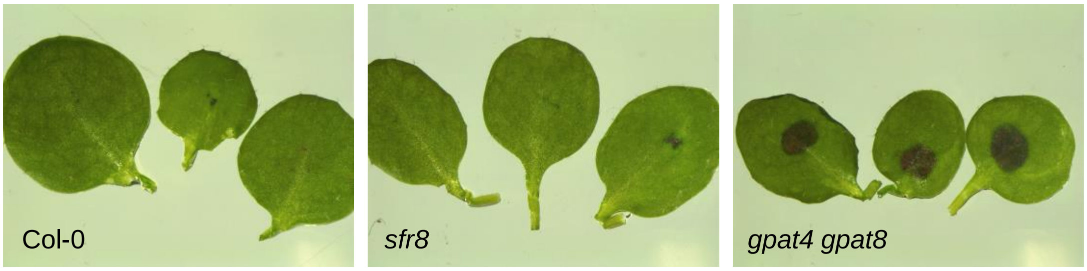

Fig.S2: Penetration of toluidine blue stain is very much lower in leaves of both wt and *sfr8* than in *gpat4gpat6*.
Cuticle permeability. A 2-µl droplet of 0.025% toluidine blue O was applied to the adaxial surface of leaves from 17-day-old plants. Images taken with Leica IC90 E camera fitted to a Leica Stereo microscope using x5 magnification show the typical range of staining patterns seen in each genotype after thorough washing in distilled water. These patterns are representative of those seen four independent tests.

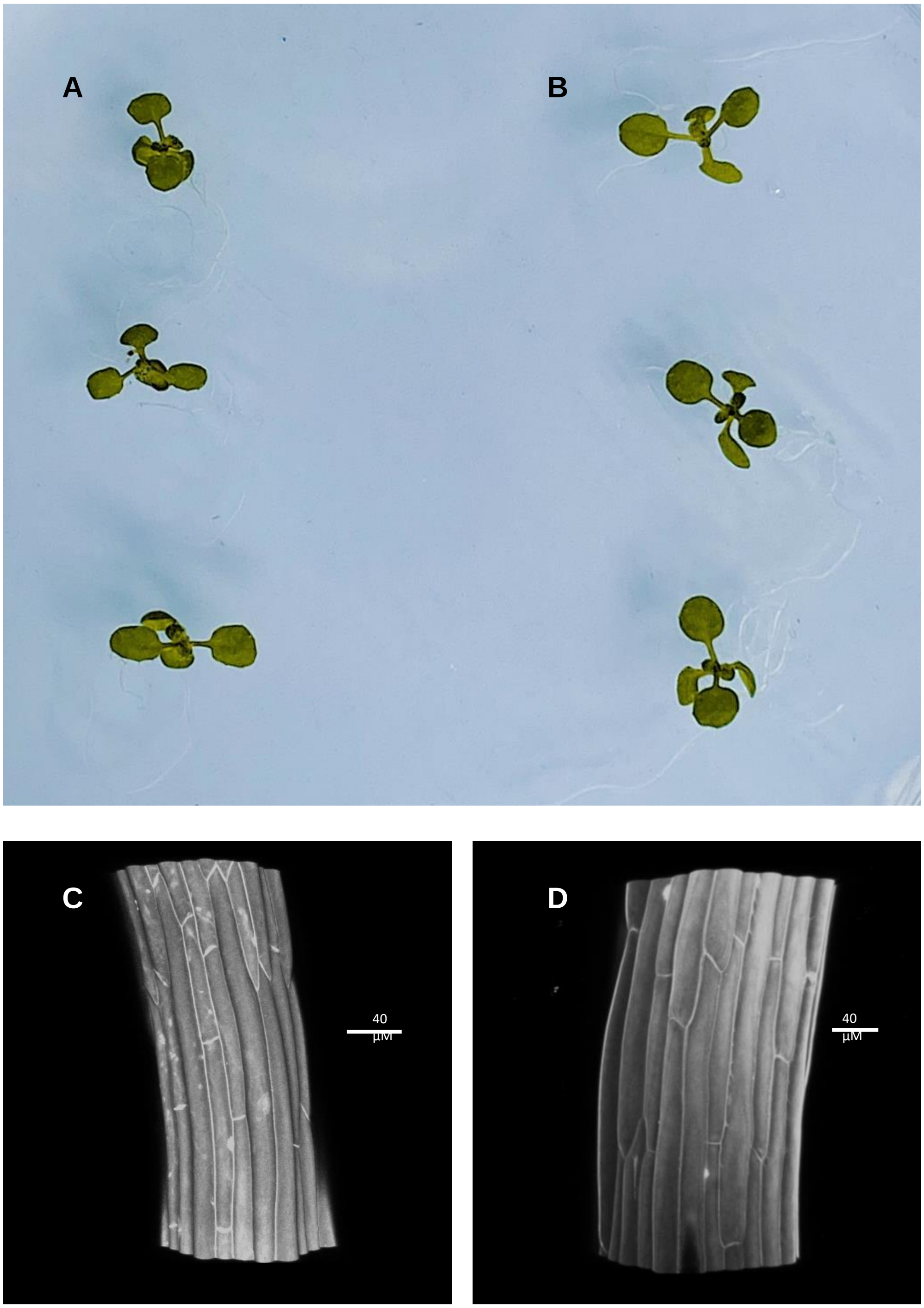

Fig.S3: *sfr8* seedlings do not show obvious signs of cell adhesion defect. Col-0 wild type (**A, C**) and *sfr8* (**B, D**). Twelve day old seedlings grown on MS agar (**A, B**). Four-day-old dark grown hypocotyls stained with propidium iodide (**C, D**). Four-day-old dark-grown hypocotyls were immersed in 0.2 mg/ml propidium iodide (Sigma) in water for 10 min and washed with water prior to imaging. Hypocotyls were placed between glass slide and coverslip separated by two further coverslips which acted as spacers to prevent tissue crushing. Samples were imaged using a ZEISS LSM 800 confocal laser scanning microscope (https://www.zeiss.co.uk) with a 20x objective lens. Propidium iodide excitation was performed using a 552 nm solid-state laser and fluorescence was detected at 600-650 nm. Images were taken as z-stacks in ZEN 2.6 Blue Edition (ZEISS; https://www.zeiss.co.uk).

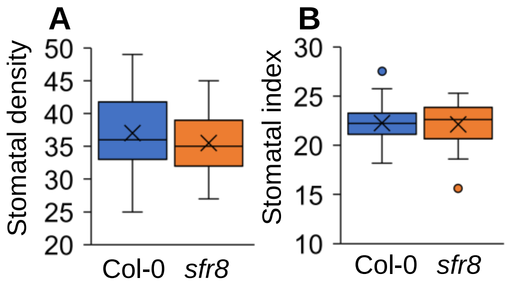

Fig.S4: Stomatal distribution is unaltered in *sfr8*. Stomatal density (number of stomata per unit area) (**A**) and stomatal index ((S/S+E)x100, where S is number of stomata per unit area and E is number of epidermal cells per unit area) (**B**). Error bars represent +/- 1 S.D. with n=24. Data analysed by two-sample t-test showed no significant differences. Stomatal index and density was calculated from counting cells of leaf peels taken from the abaxial side of the leaf.

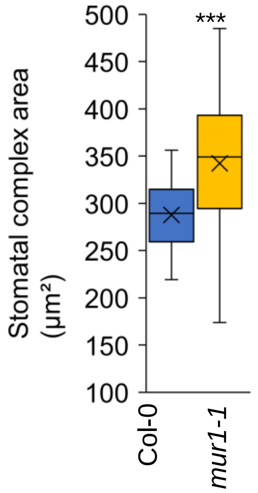

Fig.S5: Stomatal complex size in *mur1-1*. Stomatal complex area after incubation in opening buffer. Coloured box represents inter-quartile range, centre line is median, x is mean, bars are max and min values. Results are averages from two separate experiments each measuring three peels from separate plants with 10-15 stomata per peel. Data analysed by ANOVA followed by post-hoc Tukey. Results shown represent significant difference from Col-0. ***, $P<0.001$.

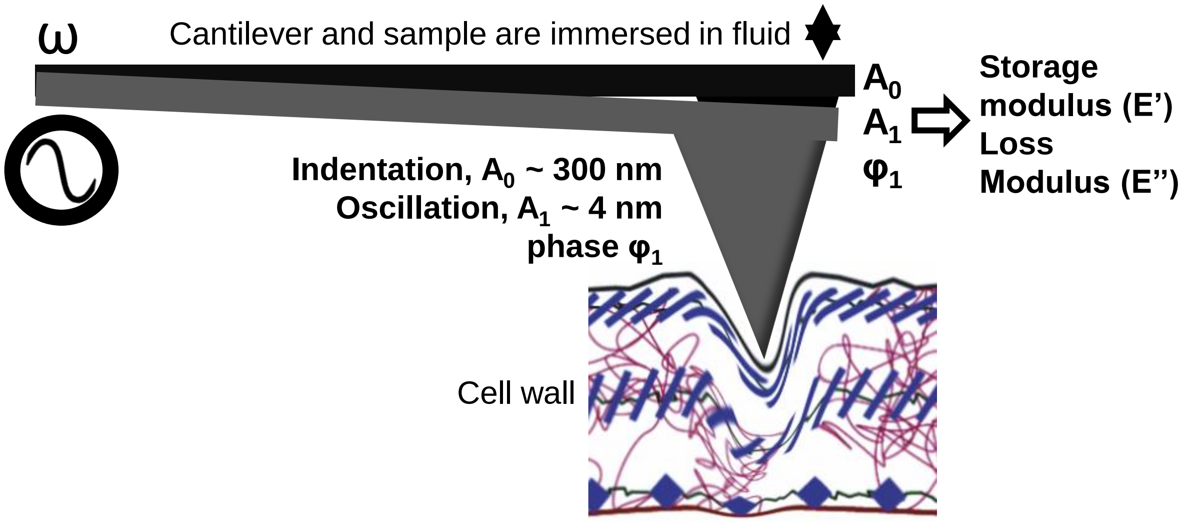

Fig.S6: Schematic representation of the multifrequency atomic force microscopy (AFM) technique used in this study, based on (Seifert *et. al.,* 2021). Reprinted from Acta Biomaterialia volume 121, Seifert J, Kirchhelle C, Moore I, Contera S. Mapping cellular nanoscale viscoelasticity and relaxation times relevant to growth of living Arabidopsis thaliana plants using multifrequency AFM. pp 371–382, Copyright (2021), with permission from Elsevier. It is a contact resonance technique, which is done by producing a constant indentation of the cell wall of the leaves of living plants in medium, and a simultaneous oscillation of the cantilever. The imaging feedback is done on the deflection of the cantilever. Using a mathematical model independent of the tip geometry it is possible to produce nm-resolution maps of the elastic modulus E' and loss modulus E''. This technique has been shown to produce quantitatively correct values of time dependent mechanical properties of plant cell walls, unlike other indentation-based techniques where the cantilever is not in constant contact with the sample as we have extensively demonstrated in Seifert *et al.,* 2021.

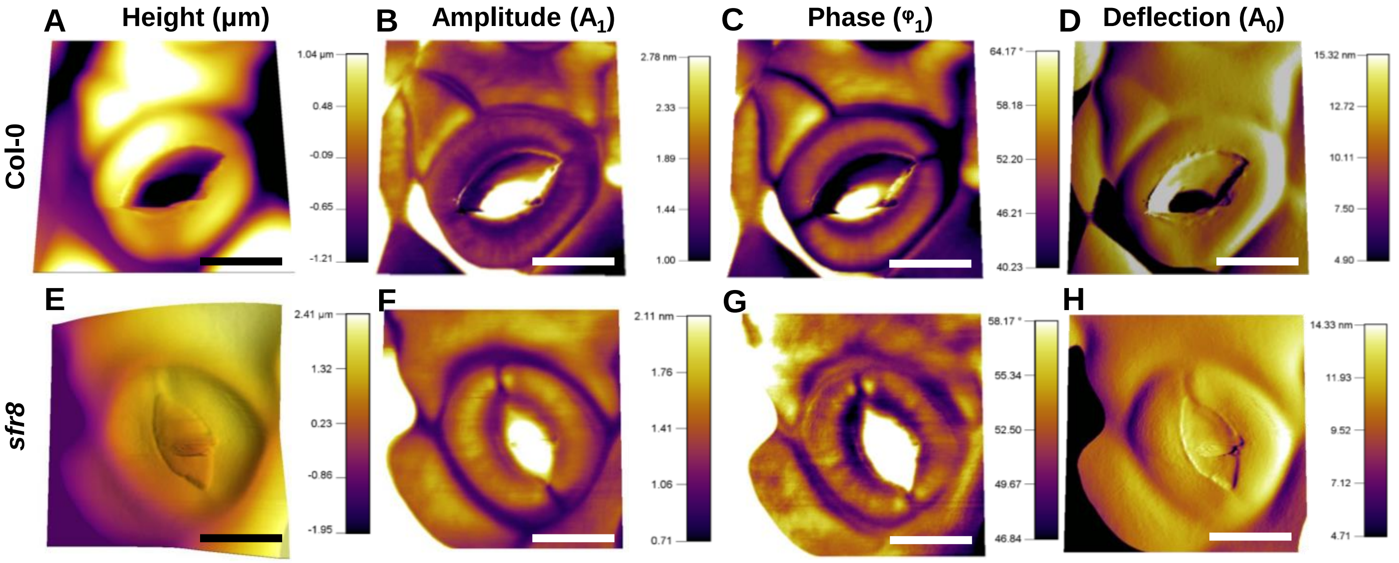

Fig.S7: Multifrequency AFM (with feedback on deflection and 1st harmonic actuated) images. (**A-D**) Col-0 and (**E-H**) *sfr8* stomata. (**A, E**) Height topography images; (**B, F**) amplitude of the first harmonic; (**C, G**) correspond to the phase images of the first harmonic; (**D, H**) deflection images. All images are 30 x 30 μm. Scale bars = 10 μm.
